# The metabolome 18 years on: a concept comes of age

**DOI:** 10.1007/s11306-016-1108-4

**Published:** 2016-09-02

**Authors:** Douglas B. Kell, Stephen G. Oliver

**Affiliations:** 1 School of Chemistry, The University of Manchester, 131 Princess St, Manchester, M1 7DN UK; 2 Manchester Institute of Biotechnology, The University of Manchester, 131 Princess St, Manchester, M1 7DN UK; 3 Centre for Synthetic Biology of Fine and Speciality Chemicals (SYNBIOCHEM), The University of Manchester, 131, Princess St, Manchester, M1 7DN UK; 4 Cambridge Systems Biology Centre, University of Cambridge, Sanger Building, 80 Tennis Court Road, Cambridge, CB2 1GA UK; 5 Department of Biochemistry, University of Cambridge, Sanger Building, 80 Tennis Court Road, Cambridge, CB2 1GA UK

**Keywords:** Metabolome, Functional genomics, Systems biology, Precision medicine

## Abstract

**Background:**

The term ‘metabolome’ was introduced to the scientific literature in September 1998.

**Aim and key scientific concepts of the review:**

To mark its 18-year-old ‘coming of age’, two of the co-authors of that paper review the genesis of metabolomics, whence it has come and where it may be going.

## Introduction

**Fig. 1:**
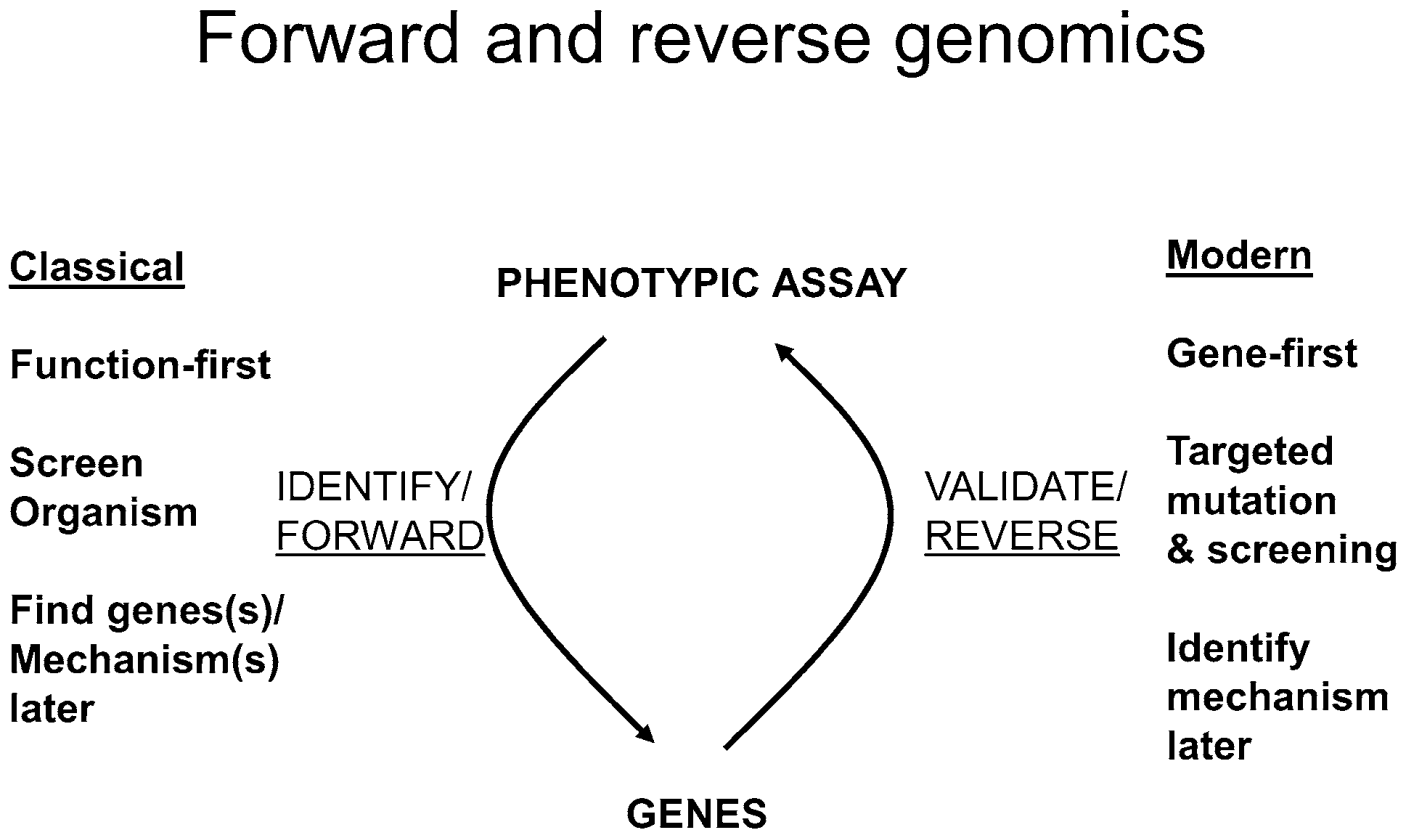
The ‘forward’ and ‘reverse’ strategies that have been used to link genes and phenotypes. Classically, one would start with a function and seek gene(s) responsible. As it became clearer that most genes were phenotypically silent, it emerged from the systematic genome sequencing programs that only a small fraction of genes had been discovered in this way. The systematic genome sequencing programs also served to change this completely, as once one ‘had’ the genes it was necessary to discover their function. A similar story can be written for drug discovery (Kell 2013)

The great advances in biology leading up to the discovery of the structure of DNA and the definition of the genetic code (Cobb 2015; Judson 1979), and the tremendous strides made since then, have been mainly pioneered by molecular genetic studies on model organisms such as *Escherichia coli* and yeasts (*Saccharomyces cerevisiae* and *Schizosaccharomyces pombe*) (Castrillo and Oliver 2004). The genius of molecular genetics lay in the design of experiments whereby fundamental theories of the workings of living cells at the molecular level could be rigorously tested by performing experiments that had a qualitative read-out (either the cells grew or they did not; either colonies were blue or they were not). This was set to change when the first chromosome sequence to be completed (that of *S. cerevisiae* chromosome III; Oliver et al. 1992) revealed that only about 20 % of the protein-encoding genes had previously been discovered by classical genetics augmented by recombinant DNA technology. It was immediately evident that the normal course of genetic research, which proceeds from mutant phenotypes to the definition of the corresponding genotype, had to be reversed. Since DNA sequencing would define all the genes, in the future we would need to move from gene to function, rather than from function to gene (Kell and Oliver 2004) (Fig. 1). This functional analysis would need to be conducted using techniques that were every bit as comprehensive as genome sequencing, and so the different levels of ’omic analysis were conceived (Oliver 1996).

Transcriptomics (the analysis of the complete complement of (m)RNA molecules in a cell, tissue, or organ) had the twin advantages of being most closely related to genomics and that it could be pursued using similar techniques—either by hybridisation of complementary nucleic acid strands or cDNA sequencing. Like the other functional’omes the transcriptome is context-dependent—it changes with the changing physiological, pathological, or developmental state of the cell. For yeast cells, the relationship between the genome and transcriptome is approximately one-to-one; introns and, therefore, differential splicing of mRNAs are rare in yeast (Hirschman et al. 2006; Stajich et al. 2007). Proteomics (Wilkins et al. 1996) (the analysis of the complete complement of protein molecules) was also context-dependent, but the relationship (even in yeast) was one-to-many due to post-translational processing and modification of the primary polypeptides generated by protein synthesis. These were the “natural” ’omes that followed from the maxim that “DNA makes RNA makes protein” (and then apparently stops), a maxim that signalled still that ‘molecular biology’ for most people meant ‘macromolecular biology’. Despite its obvious importance in biotechnology (e.g. Bu’lock 1961; Dikicioglu et al. 2013; Nielsen and Keasling 2016), metabolism was seen at that time as something of a Cinderella subject (Griffin 2006), and only a few had pioneered such analyses.

## A little pre-history

Although it was not called metabolomics, a few early workers had developed interests in using more or less comprehensive metabolic profiling systems to understand complex biological systems. Thus Williams, an early advocate of what we would now call ‘precision medicine’ (Williams 1956), recognised the potential utility of such methods, and the Hornings and their colleagues were at the forefront of instrumental implementations (Dalgliesh et al. 1966; Horning and Horning 1971). DBK carried out his D. Phil (1975–1978) in the laboratory of F. R. (‘Bob’) Whatley, whose colleague Bill Greenaway was explicitly developing GC–MS methods for the analysis of pathogenic fungi and the mode of action of fungicides. Partly because of the help of an anonymous donor with an interest in the health-giving properties of propolis (Greenaway et al. 1991), the pressure to publish then was not so intense, and this kind of work only appeared rather subsequently (Grant et al. 1988). (It was also based on a naïve interpretation of the ‘crossover theorem’ (Chance and Williams 1955), and lacked the theoretical foundations that metabolic control analysis and systems biology—see below—could provide.) At the time, much of it involved improving the reproducibility, and the production (on a 5Mbyte “Winchester” hard disk the size of a bicycle wheel) of a database of mass spectra. Plus ça change, one might say!

## The metabolome

Meanwhile, and while proteomics appeared daunting, performing functional analysis at the level of the metabolites appeared far more tractable since we calculated (wrongly, as it turned out: Jewison et al. 2012) that there were only 600–700 metabolites in the yeast cell—about an order of magnitude less than the number of protein-encoding genes (Goffeau et al. 1996). The complete complement of metabolites was also context-dependent, but there was no direct link to the genome since many genes may determine the synthesis and turnover of a single metabolite. Another major difficulty compared with the transcriptome and proteome was the recognition that the physical properties of metabolites were much more widely varied making the metabolites much more differentially extractable, and also that many were quite labile. On the other hand, the metabolic profile was directly and immediately linked to function, and potentially comprehensive methods of analysis (especially mass spectrometry and nuclear magnetic resonance) were available. Metabolic control analysis (MCA) (Fell 1992, 1996; Heinrich and Rapoport 1974; Heinrich and Schuster 1996; Kacser and Burns 1973; Kell et al. 1989; Kell and Westerhoff 1986), a precursor of modern metabolic network biology (Palsson 2006), had long explained why changes in the levels of individual genes or transcripts had relatively little effect on metabolic fluxes, but that they could necessarily—and for precisely the same reasons—have potentially very large effects on metabolite concentrations. Thus, we reasoned, also given that microbes tend to favour growth rate over growth yield (Westerhoff et al. 1983), that in order to maintain the fluxes through the metabolic networks at a relatively constant level, microbial cells would have to vary the concentrations of their constituent metabolites over a wide range—thus the concept, and the term, ‘metabolome’ was born (Oliver et al. 1998).

The initial test of the concept was pioneered in a collaborative effort between our laboratories (then in Manchester and Aberystwyth) and those of Kevin Brindle (in Cambridge) and Hans Westerhoff/Karel van Dam (in Amsterdam). The idea was that we should be able to elucidate the role of genes of unknown function by comparing the metabolomes of their deletion mutants with those of the deletion mutants of genes of known function. This concept, often called “guilt by association” (Oliver 2000), and a standard strategy in the older ‘operational fingerprinting’ (Meuzelaar et al. 1982) and the newer machine learning (Goodacre et al. 1998), was to become a prevalent one in functional genomics. In this specific example, the use of metabolomics to reveal similarities between yeast mutants was termed FANCY, for Functional ANalysis by Co-responses in Yeast, by Bas Teusink (Teusink et al. 1998)—an acronym which, for better or worse, never caught on. For all that, the concept was robustly validated by the association of the metabolomes of *pfk26* and *pfk27* deletants, and also those of a number of nuclear *petite* mutants (Raamsdonk et al. 2001; Cornish-Bowden and Cárdenas 2001). What was remarkable about this proof-of-principle study was that it worked at all, given the small number of metabolites identified in the NMR analyses. The notion that it was only necessary to monitor the most connected metabolites was tested in Kevin Brindle’s lab, using classical biochemical analyses, but this only served to emphasise the importance of using just one analytical technique to quantify all metabolites. The discriminatory power of just a limited metabolome inspired DBK to suggest monitoring the metabolites excreted into the growth medium—the metabolic ‘footprint’ or exometabolome (Allen et al. 2003, 2004; Kaderbhai et al. 2003; Kell et al. 2005), of which more later. We also recognised that Direct Injection Mass Spectrometry (DIMS) could be used to speciate intact bacterial cells (Vaidyanathan et al. 2001) and other substances (Goodacre et al. 2002), and this DIMS approach has recently been exploited to great effect by Uwe Sauer and colleagues (Link et al. 2015) to analyse the endometabolome by directly injecting living cells into a high-resolution mass spectrometer.

However, the most important outcome of this study was that metabolomics was rapidly embraced across the biological research community, and especially by plant biologists (Fiehn 2002; Fiehn et al. 2000; Jenkins et al. 2004) despite (or perhaps because of; Quanbeck et al. 2012) the fact that higher plants are considered to have the largest and most complex metabolomes in the living world. (However, we note as a caveat that most microbes have still not been brought into laboratory culture and their many secondary metabolites decrypted (Kell et al. 2015a; Lewis et al. 2010)).

## The previous 18 years

In a 2004 review (Kell 2004), one of us used the methods of text mining to analyse the areas in which metabolomics research was then most focused, identifying three main clusters: technological developments, the integration of metabolomics with other ’omics (Castrillo et al. 2007), and its use in predicting higher order properties such as disease. Shortly afterwards the Metabolomics Society and this journal were founded, with the annual meetings now attracting almost 1000 participants. The annual numbers of papers with the term metabolom* in their title or abstract continue to rise, and in 2015 amounted, at Web of Knowledge, to 3130 (in a total exceeding 18,000).

Consequently, the space available does not permit us to be even faintly comprehensive about the development of metabolomics—the papers in this journal provide an excellent starting point—but the massive improvement in mass spectrometric and chromatographic methods is clearly a huge driver (Dettmer et al. 2007; Makarov et al. 2006)) and has been so for us (e.g. (Begley et al. 2009; Dunn et al. 2011, 2015; Goodacre et al. 2004; O’Hagan et al. 2005; Zelena et al. 2009), as are improvements in mass precision and metabolite identifiability (Brown et al. 2009; Dunn et al. 2013; Kind and Fiehn 2007; Weber et al. 2011). We have also found the development of metabolic footprinting (Allen et al. 2003, 2004; Kell et al. 2005) (‘exometabolomics’) to be of value, and like many others have used both untargeted metabolomics and the related metabolic profiling (Goodacre et al. 2004) to discover new disease biomarkers (e.g. (Dunn et al. 2007; Kenny et al. 2005, 2010)).

The importance of metabolomics databases (Haug et al. 2013; Skogerson et al. 2011; Wishart et al. 2013; Zhu et al. 2013) and the need to make metabolomics data publically available (Rocca-Serra et al. 2016; Salek et al. 2015) cannot be stressed too highly.

An important trend is the use of ^13^C labelling for measuring fluxes (Zamboni et al. 2009), as well as the integration of experimental metabolomics with the genome-wide metabolic networks that are becoming available (Herrgård et al. 2008; Swainston et al. 2016; Thiele et al. 2013). Equivalently, and sadly, an important non-trend is any major improvement in the proper use of statistical and related (machine learning) methods in biological (Ioannidis 2005) and especially metabolomics (Broadhurst and Kell 2006) studies.

## Quo vadis? How will the full potential of metabolomics be revealed?

“It has been said that we always overestimate what we can do in two years and underestimate what we can do in twenty.”

P. Ball & L. Garwin (Ball and Garwin 1992)

Given the above caveat, we do not seek to be overly predictive, but some trends are obvious. The improvement in sample scale (with (Dunn et al. 2011, 2015) or potentially without (Lewis et al. 2016) the need for drift correction) is clearly one, and this will be aided by the continuing development of inter-laboratory comparisons (Abate-Pella et al. 2015) and standards for data, data analysis, and interoperability and data integration (Goodacre et al. 2007; Grapov et al. 2015; Salek et al. 2013, 2015; Sansone et al. 2007). Such things will assist greatly in the development of personalised medicine and its integration with wearable technologies. As well as the anticipated trends in sensitivity, moving towards the necessary single-cell analyses, it is clear that many more metabolites remain to be discovered, even in simple hosts (Carbonell et al. 2013, 2014) (probably as a result of enzyme promiscuity Currin et al. 2015; Jeffryes et al. 2015). Such analyses are greatly aided by the use of proper descriptors of small molecule structures, such as SMILES (Weininger 1988) and InChI (Coles et al. 2005; Heller et al. 2013; Spjuth et al. 2013), that allow cheminformatic reasoning about properties such as drug-metabolite similarities (Dobson et al. 2009b; O’Hagan and Kell 2015b, O’Hagan and Kell 2016; O’Hagan et al. 2015).

Another trend will be further automation of instrument tuning (Bradbury et al. 2015), non-invasive methods (Rattray et al. 2014), and an increased portability of instrumentation such that it may even be used in the field (as is now the case for genomics (Ashton et al. 2015; Kilianski et al. 2015) and biometrics). This is clearly assisted by ‘ambient mass spectrometry’ (Cooks et al. 2006), and the impressive ‘iKnife’ (Alexander et al. 2016; Balog et al. 2013) pioneering of such measurements in the operating theatre. This kind of development will be especially important in terms of environmental metabolomics (Bundy et al. 2009) and the ‘exposome’ (the integrated load of xenobiotics that an individual has accumulated in his/her lifetime) (Athersuch and Keun 2015; Rappaport et al. 2014). The extensive data that will be generated will be harvested via the ‘Internet of Things’ (Ellis et al. 2015), scientific reasoning will be further automated (King et al. 2004, 2009; Williams et al. 2015), and in an era where the methods of ‘artificial intelligence’ are starting to show human-level abilities, at least in restricted domains (Koza 2010; Mnih et al. 2015; Silver et al. 2016), we shall be wise to exploit such methods.

At least as judged by their appearance in the literature, some enzymes in a given organism are much more greatly studied than are others, a phenomenon referred to as ‘publication asymmetry’ (César-Razquin et al. 2015). As assessed in that paper (César-Razquin et al. 2015), solute carriers (SLCs (Hediger et al. 2004)) or transporters are the most neglected group of genes in the human genome. Our own analyses also point up their major importance in flux control (Walter et al. 1987), drug transport (Dobson et al. 2009a; Dobson and Kell 2008; Kell 2013, 2015a, 2015b, 2016; Kell et al. 2013, 2011; Kell and Oliver 2014; Lanthaler et al. 2011; Mendes et al. 2015; O’Hagan and Kell 2015a) and biotechnology (Kell et al. 2015b). Thus we consider that, although challenging, compartment-based metabolomics, where such transporters are necessarily involved, is likely to become a substantial field of itself. Indeed, our improved understanding of a special compartment called the microbiome shows that not all of the genes and metabolites involved in supposedly non-communicable diseases even arise from the host (Honda and Littman 2016; Potgieter et al. 2015; Wang et al. 2011; Wikoff et al. 2009).

Biological studies will be much aided by the ability to manipulate genomes at will. Henrik Kacser, as a major part of his motivation for developing MCA in the first place, had long ago explained why much more sensitive analyses are possible with haploids than with diploids (Kacser and Burns 1981). Thus, a particularly nice example was given by the work of Superti-Furga and colleagues (Winter et al. 2014) on a near-haploid cell line showing that at least 99.5 % of the uptake of the drug sepantronium bromide proceeded through a specific transporter, and thus that any transbilayer flux was negligible.

The original paper (Oliver et al. 1998) concluded “many of these techniques are sufficiently general that, once they have been tried and tested in the experimentally tractable yeast system, they should be directly applicable to the study of the functional genomics of higher organisms”. Certainly this has been borne out, and overall, then, metabolomics has had a very healthy childhood and adolescence. Perhaps now the exposome, and even more comprehensive studies, will usher in the (for us much-vaunted (Kell 2004, 2006; Kell et al. 2005) but largely awaited) integration of metabolomics and systems biology. If it does, it will have been well worth the wait.
